# In vivo free-breathing DTI & IVIM of the whole human heart using a real-time slice-followed SE-EPI navigator-based sequence: a reproducibility study in healthy volunteers

**DOI:** 10.1186/1532-429X-17-S1-P383

**Published:** 2015-02-03

**Authors:** Kévin Moulin, Pierre Croisille, Thorsten Feiweier, Benedicte M Delattre, Hongjiang Wei, Benjamin Robert, Olivier Beuf, Magalie Viallon

**Affiliations:** 1 Siemens Healthcare France, Paris, France; 2 CREATIS; CNRS (UMR 5220); INSERM (U1044); INSA Lyon; Université de Lyon, Lyon, France; 3 Healthcare, Siemens AG, Erlangen, Germany; 4 Departement of Radiology, Centre Hospitalier Universitaire de Saint-Etienne, Université Jean-Monnet, Saint-Etienne, France

## Background

In vivo cardiac diffusion using either the Intra-Voxel Incoherent Motion (IVIM) model or the Diffusion Tensor Imaging (DTI) model shows promise to provide new insights into cardiac disease processes. However, due to the combined challenge of respiratory and heart motion [1,2], currently proposed acquisition methods may not be applicable in patients due to long acquistion time. In this study, we propose to use the motion information provided by a navigator to prospectively update in real time the position of the diffusion weighted slices, offering an efficient free-breathing strategy for rapid and improved cardiac diffusion acquisition using a single-shot spin-Echo EPI sequence.

## Methods

The proposed technique was performed on 10 volunteers on 1.5T scanner to quantify the reproducibility of the diffusion parameters (*FA*, *MD*, *f*, *D* and D*). Double oblique short-axis DW images were acquired with a 128 × 80 pixel matrix and rectangular FoV of 350 × 220mm2. Short axis DW-slices (6-mm-thick) were obtained with an in-plane resolution of 2.7 × 2.7mm2 interpolated in-plane to 1.35 x 1.35mm2; the bandwidth was 2442Hz/pixel, and GRAPPA factor 2 was used. Multiple TDs shifted every 10ms were acquired for PCAtMIP reconstruction. DTI acquisitions parameters were: interleaved 10 contiguous slices; TE=45ms; TR= number of slices x heart rate (e.g., 10s for a heart rate equal to 60 bpm); and 12 diffusion directions with a b-value of 350s/mm2 and 10 TDs for a total acquisition time of 22min. The IVIM acquisition parameters were 5 interleaved slices with a gap of 100%; TE=42ms; TR= 5s; 6 diffusion directions with b-values 0, 15, 30, 50, 75, 100, 200, 300, 400s/mm2 and 5 TDs were acquired for total time of 20min. Inter-measurement reproducibility among repeated measures was assessed by the Lin concordance coefficient (LinCC) and the complementary Bland and Altman method with LinCC coefficient ρ_c_, r (a measure of precision) and C_b_ (a measure of accuracy).

## Results

With a 100% breathing cycle scanning efficiency, the slice-following strategy is a powerful head-foot respiratory motion management solution for SE-EPI cardiac diffusion Vascular fraction *f* and the diffusion coefficients *D* and D* were determined to be 35.6±11.9x10^-3^mm2/s for D*, 1.23±0.19x10^-3^mm2/s for *D* and 0.115±0.017 for *f*. From the DTI protocol, the Mean Diffusivity (*MD*) was 1.57±0.13x10^-3^mm2/s and the Fractional Anisotropy (*FA*) 0.35±0.04. *MD* (*ρ_c_*=0.69,*r*= 0.70,*C_b_*=0.99), *D* (*ρ_c_*=0.92, *r*=0.99, *C_b_*=0.93) and *f* (*ρ_c_*=0.69, *r*=0.75, *C_b_*=0.91) was highly reproducible exept for D* (*ρ_c_*=-0.214, *r*=-0.229, *C_b_*=0.93) and *FA* (*ρ_c_*=0.075, *r*=0.12, *C_b_*=0.56) but which remained accurate.

## Conclusions

The slice-followed SE-EPI cardiac diffusion sequence is a promising solution for clinical implementation, allowing unprecedent acquisition speed that could be tailored to specific needs.

## Funding

N/A.

**Figure 1:**
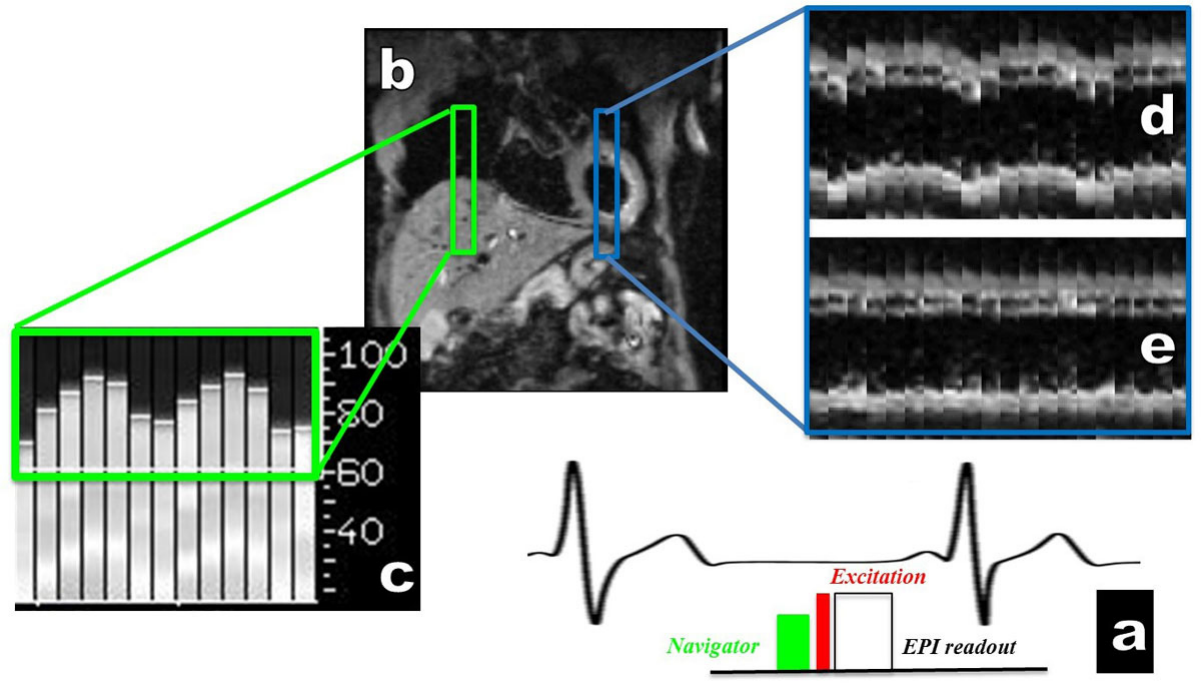
**a) A pencil beam navigator is played out before each acquisition and placed on the top of the liver b). The information given by the navigator c) is used, through a tracking factor of 0.6, to move the slice in real time during the breathing cycle.** Performance of the respiratory head-foot motion correction for SE-EPI diffusion with slice following was evaluated with coronal b-values=30 s/mm2 image from a series of diffusion-weighted images acquired over 1 min. d) and e) breathing coronal acquisition without and with slice following.

**Figure 2:**
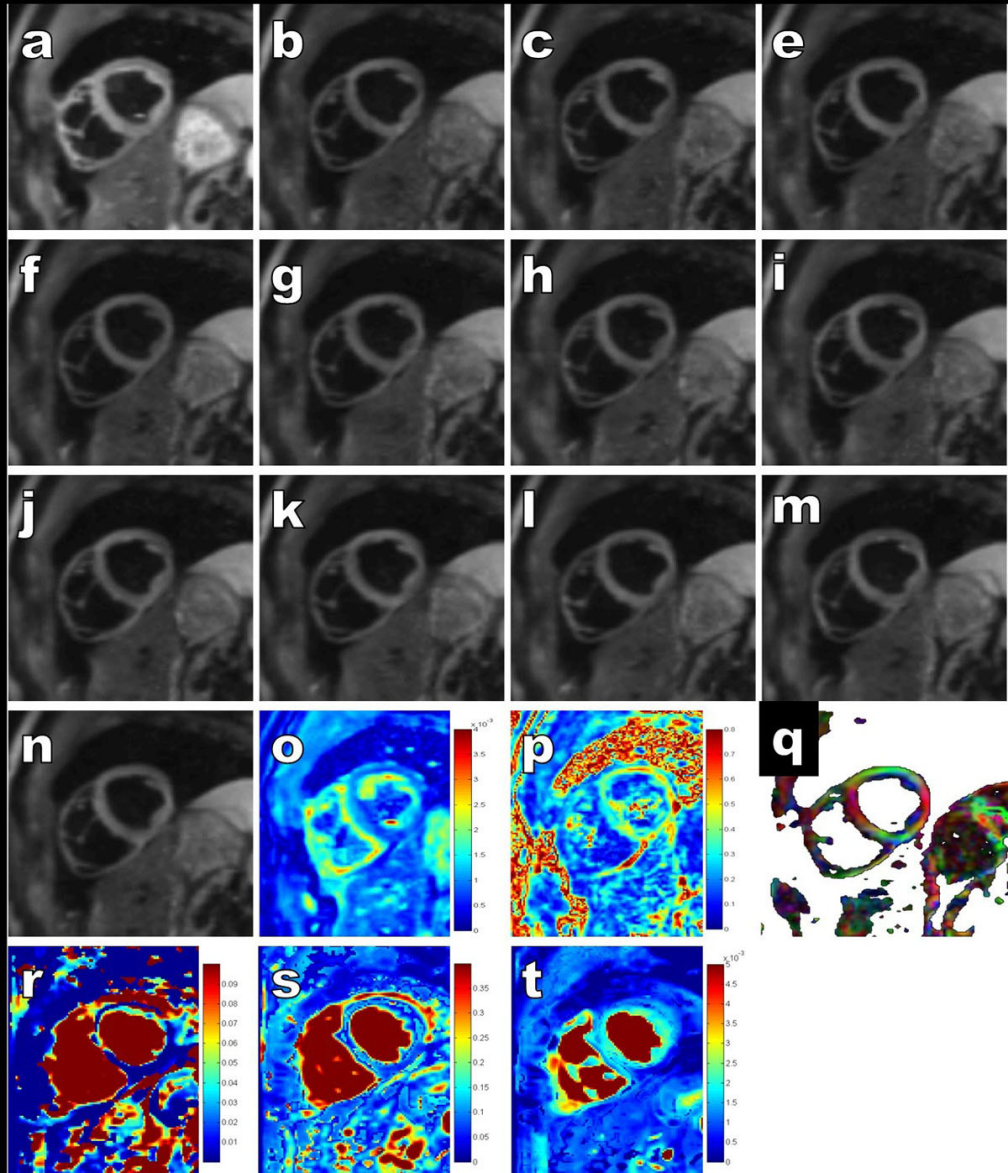
Example of DTI raw images of mid-ventricular short axis slices: a) b-value=0 image (with dark blood ~= 5 s/mm2) b-n) b-values=350 s/mm2 for 12 directions and calculated parametric maps: o) mean diffusivity *MD*, p) fraction of anisotropy *FA*, q) colored *FA* maps displaying the classical fiber orientation; parametric maps from IVIM dataset : *r*) fast diffusion D*, s) perfusion fraction *f*, and t) diffusion D.
